# Bone marrow-derived macrophages from a murine model of Sjögren's syndrome demonstrate an aberrant, inflammatory response to apoptotic cells

**DOI:** 10.1038/s41598-022-12608-4

**Published:** 2022-05-21

**Authors:** Richard Witas, Yiran Shen, Cuong Q. Nguyen

**Affiliations:** 1 Department of Infectious Diseases and Immunology, College of Veterinary Medicine, University of Florida, PO Box 110880, Gainesville, FL 32611-0880 USA; 2 Department of Oral Biology, College of Dentistry, University of Florida, Gainesville, FL USA; 3 Center of Orphaned Autoimmune Diseases, University of Florida, Gainesville, FL USA

**Keywords:** Immunology, Rheumatology

## Abstract

Sjögren's syndrome (SjS) is a female-dominated autoimmune disease involving lymphocytic infiltration of the exocrine glands. We have previously demonstrated cleavage of the TAM (Tyro3, Axl, Mer) receptor Mer is enhanced in SjS, leading to defective efferocytosis. Mer also plays a role in modulating phagocyte inflammatory response to apoptotic cells. Here we investigated the SjS macrophage response to apoptotic cells (AC). Bone marrow-derived macrophages (BMDMs) from SjS-susceptible (SjS^s^) C57BL/6.NOD-*Aec1Aec2* mice and C57BL/6 (B6) controls were treated with either AC or CpG-oligodeoxynucleotides. RNA was collected from macrophages and bulk sequencing was performed to analyze transcripts. Cytokine expression was confirmed by Bio-plex. RT-qPCR was used to determine toll-like receptor (TLR) 7 and 9 involvement in BMDM inflammatory response to apoptotic cells. SjS^S^ BMDMs exhibited a distinct transcriptional profile involving upregulation of a broad array of inflammatory genes that were not elevated in B6 BMDMs by AC. Inhibition of TLR 7 and 9 was found to limit the inflammatory response of SjS^S^ BMDMs to ACs. ACs elicit an inflammatory reaction in SjS^S^ BMDMs distinct from that observed in B6 BMDMs. This discovery of aberrant macrophage behavior in SjS in conjunction with previously described efferocytosis defects suggests an expanded role for macrophages in SjS, where uncleared dead cells stimulate an inflammatory response through macrophage TLRs recruiting lymphocytes, participating in co-stimulation and establishing an environment conducive to autoimmunity.

## Introduction

Sjögren's syndrome (SjS) is estimated to be the second most common autoimmune disease after rheumatoid arthritis (RA) and reportedly affects 0.7% of the population of European Americans^1^. SjS is among the most female-dominated autoimmune diseases known to affect women in a 9:1 ratio^2^. It is characterized by an autoimmune attack of the salivary and lacrimal glands resulting in dry mouth and dry eyes. Similar to systemic lupus erythematosus (SLE), SjS is characterized by upregulation of interferon (IFN) stimulated genes, known as an IFN signature. The type I IFN signature is believed to predominate in SjS patient peripheral blood. It has been reported to affect 53–81% of SjS patients depending on the patient populations in different studies^3,4^. The type II IFN signature is less well studied but has been described to predominate in SjS patient minor salivary gland^4^. A recent study integrated multi-omics data from 304 primary SjS patients compared to 330 healthy controls in order to classify SjS patients into subgroups, thus providing a means to re-evaluate clinical trial results in the context of more homogenous clusters of patients with similar disease phenotypes^5^. Interestingly, the degree of IFN signature gene enrichment, and whether a type I or type II IFN signature was dominant were two major features that distinguished the four patient subgroups identified in this study^5^.

Furthermore, both type I and type II IFN signaling have been proven to be necessary for developing disease in the SjS-susceptible mouse model (SjS^S^) C57BL/6.NOD-*Aec1Aec2*^6,7^. These findings indicate the critical role inflammatory signaling plays in driving autoimmunity in SjS. However, the factors driving this inflammatory state remain uncharacterized. Studies into the genetic risk factors of SjS have identified an association with factors involved in controlling inflammation, including signal transducer and activator of transcription (*STAT)4*, interleukin (*IL)12A*, and interferon-regulatory factor (*IRF)5* suggesting a role for dysfunctional inflammatory regulation in the disease^8^.

Aberrant disposal of dead cells has been previously described as a contributing factor to inflammation in SjS^9^. Under homeostatic conditions, apoptotic cells are disposed of by phagocytes, particularly macrophages. The TAM receptor tyrosine kinase Mer on the surface of macrophages plays a critical role in facilitating efferocytosis^10^. The TAM receptors comprise a family of three receptor tyrosine kinases Tyro3, Axl, and Mer. The TAMs possess an extracellular immunoglobulin-like N-terminus that indirectly recognizes phosphatidylserine on the surface of apoptotic cells through bridging molecules such as Gas6 and ProteinS. The TAMs also possess an intracellular tyrosine kinase domain at their C-terminus enabling signaling through the receptor which can culminate in multiple outcomes including phagocytosis of apoptotic cells, regulation of inflammation, and proliferation^11^.

It has previously been established that Mer can be cleaved from the cell surface, inactivating it and impairing signaling through the Mer pathway, thereby decreasing Mer-mediated signaling outcomes such as efferocytosis^12,13^. We recently demonstrated that SjS patients and SjS^S^ mice exhibit enhanced cleavage and inactivation of Mer. The lack of functional Mer impaired Rac1 activation and subsequent efferocytosis in SjS^S^ bone marrow-derived macrophages (BMDMs)^14^. Mer is also involved in negatively regulating inflammatory signaling through the induction of suppressors of cytokine signaling (SOCS1) and SOCS3^11^. Furthermore, apoptotic bodies can progress to secondary necrosis and release self-antigens or damage associated molecular patterns (DAMPs) when not cleared away adequately. These newly released DAMPs can bind and activate the immune system's toll-like receptors (TLRs) signaling pathway. The unregulated activation of TLR signals can promote autoimmunity^15^.

In light of this information, in this study, we sought to investigate the immunological consequences of macrophages in response to apoptotic cells (ACs) in SjS^S^ mice, which have been shown to exhibit impaired Mer signaling. We observed that SjS^S^ BMDMs demonstrated a highly inflammatory response to ACs in comparison to B6 controls consisting of the upregulation of multiple inflammatory cytokines, macrophage activation genes, and co-stimulatory molecules. We hypothesized that the SjS^S^ BMDM response to ACs was a result of delayed clearance of ACs resulting in spillage of intracellular contents and activation of macrophage TLRs. We observed that the SjS^S^ BMDM inflammatory response to ACs was abrogated by TLR7 and 9 inhibition.

## Methods

### Mice

C57BL/6 (B6) and B6J.NOD/ShiLtJ-*Aec1Aec2* (B6.NOD-*Aec1Aec2)* mice were bred and maintained under specific pathogen-free conditions in the animal facility of Animal Care Services at the University of Florida. All animals were maintained on a 12-h light–dark schedule and were provided with food and water ad libitum. Mice were anesthetized with isoflurane and euthanized by cervical dislocation, and their bone marrow and thymocytes were freshly harvested for analysis. All mice included in this study were female. The University of Florida's Institutional Animal Care approved all protocols respective to breeding and the use of animals described herein. The experimental methods were carried out in accordance with the appropriate approvals and relevant guidelines.

### BMDM isolation

BMDMs were grown, as described previously^16^. Briefly, 7–12-week-old B6 and SjS^S^ mice were euthanized, and bone marrow cells were collected by flushing the femurs and tibias with RPMI (Lonza, Allendale, USA) complete media containing 10% FBS, 1% penicillin/ streptomycin, 2 mM of L-glutamine, 0.05 mM of β-mercaptoethanol supplemented with 20% L929 supernatant containing M-CSF and L929 media preparation, as described^16^. Cells from each mouse were filtered through a 70-μm cell strainer and plated in untreated Petri dishes with 5 million cells per dish. Cells were maintained in a 37 °C incubator for seven days with 50% media changes performed every two days. When differentiation was completed after seven days, macrophages were detached from plates by 15-min incubation with Accutase (Innovative Cell Technologies, San Diego, USA) on ice. Cells were counted and redistributed to 24-well plates with 3 × 10^5^ cells per well, and L929 supplementation was reduced to 5% at 24 h before treatment. An example of macrophage purity at seven days is included in Supplementary Figure 1 obtained using a BD Fortessa flow cytometer (BD, Franklin Lakes, USA) with CD11b (Biolegend, San Diego, USA) and F4/80 (Biolegend) staining.

### Thymocyte collection and apoptosis

Thymocytes was used as the cell source for apoptotic cells in this study due to the following reasons: (1) the process of inducing apoptosis in thymocytes by dexamethasone is well established via DNA fragmentation and cell death through the diacylglycerol generation and G-protein-dependent phosphatidylinositol-specific phospholipase C and eventual activation of an acidic sphingomyelinase, (2) thymocytes are readily acquired in large numbers which are needed for the assays, and (3) under basal conditions, macrophages readily efferocytose apoptotic thymocytes, allowing for examination of efferocytic defects^17,18^. To obtain thymocytes, thymi were collected from 7 to 12-week-old B6 and SjS^S^ mice and each thymus was cut into small pieces using scissors and processed separately. Thymi were incubated in an enzymatic solution containing RPMI, 1 mg/mL of collagenase IV (Sigma Aldrich, Saint Louis, USA), and 1 mg/mL of DNase I (Sigma Aldrich) at 37 °C for 20 min. The supernatant containing thymocytes was collected on ice in RPMI with 10% FBS. Fresh enzyme solution was added to the individual thymi and agitated using a 5-mL pipette before incubating for an additional 15 min. The process was repeated with an 18-G needle for mechanical agitation. The process was repeated using a 25-G needle, and then the supernatant was passed through a 70-μm cell strainer before the cells were washed and counted. Thymocytes were rendered apoptotic by a 4-h incubation with 2 μM of dexamethasone (Tocris, Bristol, UK) at 37 °C in RPMI supplemented with 1% FBS. Thymocytes were washed twice in PBS and were determined to be apoptotic by propidium iodide (Biolegend) staining as shown in Supplementary Figure 2A, B.

### BMDM AC treatment and RNA collection

Apoptotic thymocytes were incubated with macrophages in a 5:1 ratio for 2 h at 37 °C. Alternatively, BMDMs were treated with 1 μM CpG-ODN (InvivoGen San Diego, USA) for 6 h. Both treatments were performed in RPMI complete with heat-inactivated FBS. After treatment, BMDM were washed with PBS three times to remove apoptotic cells and incubated an additional 4 h in complete RPMI to allow for transcriptional changes in BMDMs and digestion of thymocyte RNA. BMDMs were washed with PBS before BMDM RNA was collected using a RNeasy kit (Qiagen, Hilden, Germany) according to manufacturer instructions. RNA concentration and 260/280 ratio were determined using a spectrophotometric plate-reader (Tecan, Mannedorf, Switzerland) and samples were kept at -80 °C until use.

### RNA-Seq

RNA sequencing was performed at the University of Florida Interdisciplinary Center for Biotechnology Research using Illumina NovaSeq S4 (Illumina, San Diego, CA, USA) sequencing by applying standard manufacturer's protocols. All samples passed quality controls and about 60 million 101 bp paired-end reads were generated for each sample.

### Differentially expressed gene and pathway analysis

Partek Flow software (Partek, St. Louis, USA) was used to align samples to the mouse mm10 genome using the STAR alignment tool. Following alignment, reads were quantified to the mm10 annotation model using the Partek E/M annotation tool. Differentially expressed features were determined by running normalized reads through the Partek GSA tool. The read counts for each sample were used for differential expression analysis with the DESeq2 (version 3.13) package (https://doi.org/10.1186/s13059-014-0550-8). The differentially expressed genes were identified using adjusted p-value < 0.05 and log2 fold change > 1.3. Principle component analysis (PCA) showing samples variance was carried out using the built-in package function, plot PCA. Heatmaps were created using pheatmap (version 1.0.12) package in R. The complete list of identified genes was used to generate volcano plots in R. For heatmap graphs and enrichment pathway analysis, the variance of genes across all phenotypes and treatment was calculated, the top-20 highly variable upregulated genes, manually chosen inflammatory-related genes were used for further analysis.

Metascape (http://metascape.org) was utilized for gene function annotation and enrichment pathway analysis by default setting. Metascape provides a workflow integrating gene annotation, membership analysis, and multi-gene-list meta-analysis. The input genes are searched and compared in different online experimental validated databases in order to determine if a set of genes participates in certain molecular pathways related to metabolic or physiological functions^19^.

### Bio-Plex evaluation of cytokine production

Syngeneic apoptotic thymocytes were incubated with BMDMs in a 5:1 ratio in 500µL for 24 h at 37 °C. Treatment was performed in RPMI complete with heat-inactivated FBS. After treatment, media was collected, aliquoted, and stored at − 80 °C until use. Cytokine levels were evaluated using a BioPlex Pro Mouse Cytokine Th1 Panel kit (Bio-Rad, Hercules, USA). Media samples were centrifuged before use, 50μL of samples was used undiluted according to manufacturer protocol. Experimental results were acquired using the Bio-Rad Magpix system. The data were analyzed using the Bio-Plex Manager software (Bio-Rad).

### Efferocytosis assay with TLR stimulation and inhibition

Thymocytes were collected as above; however, to simulate necrotic conditions, thymocytes were killed by heating to 56 °C for 20 min, followed by two-hour incubation at 37 °C in PBS with 1% FBS. BMDM treatment was conducted as follows: Control condition received no TLR stimulation or apoptotic cells. TLR7a (agonist) condition was treated with 30 µg/mL imiquimod (InvivoGen) for 6 h. TLR9a condition was treated with 1 µM CpG (InvivoGen) for 6 h. Macrophage survival of imiquimod treatment is demonstrated in Supplementary Figure 2C. BMDMs were treated with apoptotic cells for 2 h in the AC treated condition, followed by BMDMs being washed three times, and incubated for an additional 2 h. TLR7i (inhibitor) condition was treated with 5 µM TLR7 inhibitor ODN 20,958 (MACS Miltenyi Auburn, USA) for 2 h prior to AC addition. The inhibitor was re-added during 2 h AC treatment. Following AC treatment, the BMDMs were washed three times to remove apoptotic cells and incubated for an additional two hours. TLR9i condition was treated with 1 µM TLR9 inhibitor ODN 4084-F (InvivoGen) for 2 h prior to AC addition. The inhibitor was re-added during 2 h AC treatment. The BMDMs were washed three times to remove apoptotic cells and incubated for an additional two hours. RNA and protein were isolated from BMDMs following treatment using a ThermoFisher PARIS (protein and RNA isolation) kit according to manufacturer protocol. A Bio-Rad RT qPCR 1 step kit (Bio-Rad) was used to measure the expression of *Ifnb, Ifng, Il6, Il1b, Il12b, Il10*, and *Mer* with *Hprt* as a reference gene according to manufacturer protocol. Primer sequences are included in Table 1. RT qPCR data were analyzed using CFX Maestro software (Bio-Rad), and expression was reported as a relative normalized expression.

**Table 1:** RT qPCR primer list.

Gene name	Forward primer	Reverse primer
*Ifnb*	AAG AGTTACACT GCCTTTGCCATC	CACTGTCTGCTG GTGGAGTTCATC
*Ifng*	AGCGGCTGACTGAACTCAGATTGTAG	GTCACAGTTTTCAGCTGTATAGGG
*Il6*	GATGGATGCTACCAAACTGGA	CCAGGTAGCTATGGTACTCCAGAA
*Mer*	ACCTCCACACCTTCCTGTTA	CACACATCGCTCTTGCTGGT
*Il1b*	AAGGGCTGCTTCCAAACCTTTGAC	ATACTGCCTGCCTGAAGCTCTTGT
*Il12b*	ATGAGAACTACAGCACCAGCTTC	ACTTGAGGGAGAAGTAGGAATGG
*Il10*	ATTTGAATTCCCTGGGTGAGA	CACAGGGGAGAAATCGATGACA
*Hprt*	AGTGTTGGATACAGGCCAGAC	CGTGATTCAAATCCCTGAAGT

### Statistical analyses

Statistical evaluations were determined using one-tailed Mann–Whitney t-tests or ANOVA analyses where appropriate, all generated by the GraphPad Prism 8 software (GraphPad Software, La Jolla, USA). In all cases, *p* values < 0.05 were considered significant.

### Adherence to ARRIVE guidelines

The reporting in the manuscript follows the recommendations in the ARRIVE guidelines for animal research.

## Results

### SjS^S^ BMDM response to apoptotic cells is transcriptionally distinct from control BMDMs

We previously described enhanced Mer inactivation and decreased efferocytosis in BMDMs and resident peritoneal macrophage of the C57BL/6.NOD-*Aec1Aec2* (SjS^S^) mouse model^14^. The SjS^S^ mouse was generated by breeding two intervals influencing susceptibility to diabetes (Idd), I*dd3* (*Aec1*) and *Idd5* (*Aec2*) from the non-obese diabetic (NOD) mouse onto a C57BL/6 (B6) background, thus establishing a mouse line that exhibited exocrine gland pathology without diabetes^20^.

The objective of this study was to understand if the decreased Mer signaling described previously compromised the typical anti-inflammatory response of macrophages to ACs within the SjS^S^ model. RNA-seq was performed to assess BMDM response to ACs. B6 and SjS^S^ BMDMs were differentiated and incubated with dexamethasone-killed apoptotic thymocytes for two hours. Upon the conclusion of the incubation period, BMDMs were washed three times to remove un-engulfed ACs and incubated an additional three hours to allow for transcriptional changes in the BMDMs and degradation of thymocyte RNA. Thymocyte RNA's degradation was confirmed by evaluating the thymocyte-specific genes *Cd3* and *Trac*, which were not detectable in BMDM samples. As a control for an alternate inflammatory stimulus, BMDMs were treated with CpG to stimulate TLR7 signaling.

**Figure 1:**
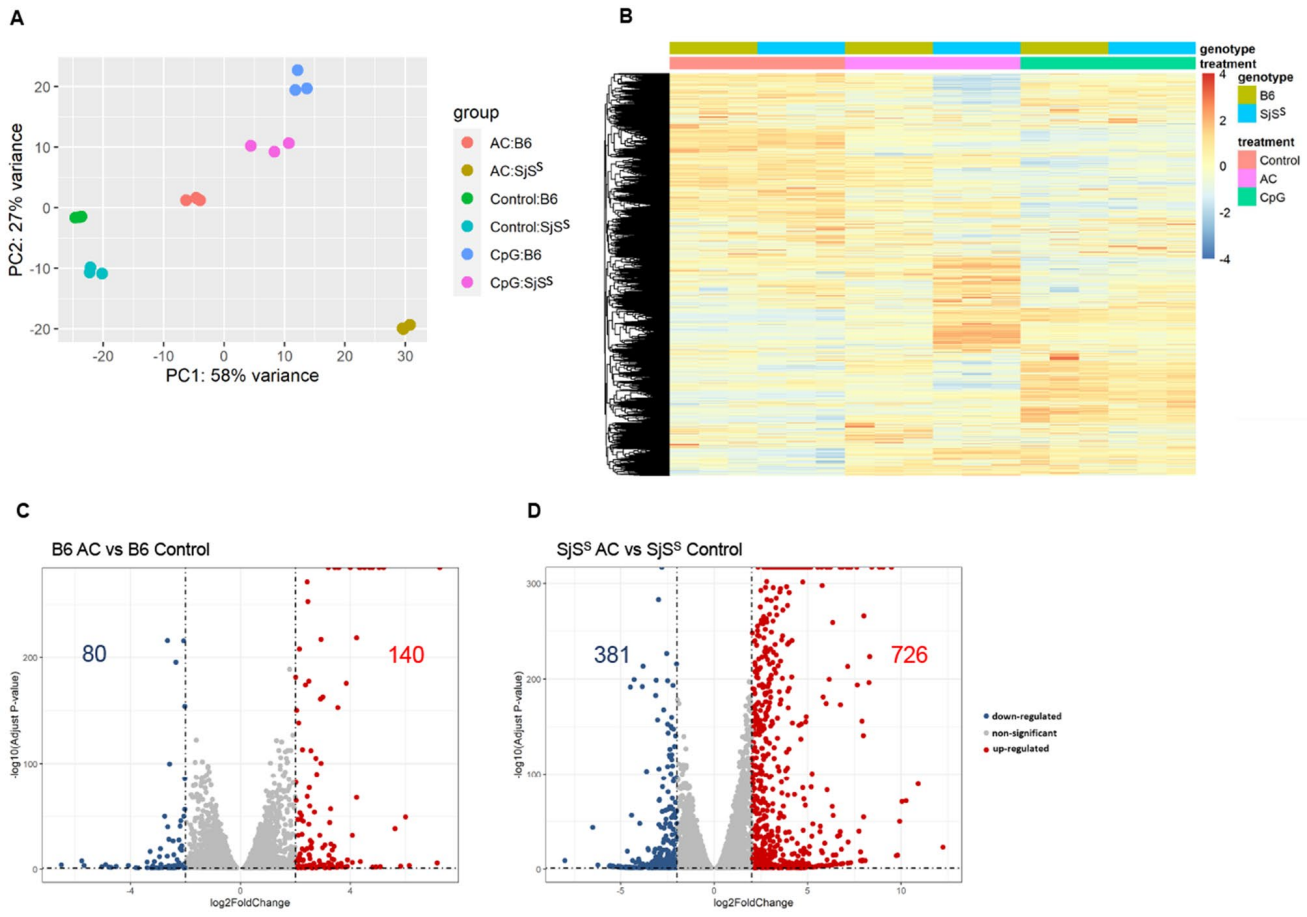
Efferocytosis induces distinct transcriptional profiles in B6 and SjS^S^ BMDMs. BMDMs were cultured from B6 (n = 3 mice) and SjS^S^ mice (n = 3 mice) and untreated or treated with CpG or apoptotic cells (AC). Following incubation, cells were washed three times and incubated an additional 3 h before RNA was collected for RNA-Seq. (**A**) Principal component analysis (PCA) plot of whole transcriptome RNA Seq data Control B6 (green), Control SjS^S^ (teal), CpG B6 (blue), CpG SjS^S^ (purple), AC B6 (red), and AC SjS^S^ (gold) (n = 3). (**B**) Heatmap of differentially expressed genes between Control B6, Control SjS^S^, CpG B6, CpG SjS^S^, AC B6, and AC SjS^S^ conditions. (**C,D**) Volcano plot of differential gene expression between Control and AC treatments for B6 (**C**) and SjS^S^ (**D**). Upregulated genes with fold change ≥ 1.3 and *P* < 0.05 are in red, and down-regulated genes with fold change ≤ 1.3 and *P* < 0.05 are in blue. Several significantly altered genes are indicated.

Tight clusters of triplicates for all conditions were observed under principal component analysis (PCA); however, AC treated B6 and SjS^S^ BMDMs exhibited differing transcriptional responses (Fig. 1A). This was in contrast to CpG treated B6 and SjS^S^ samples, which demonstrated closer clusters than observed in AC treatment. A heat map constructed for all samples indicated minor differences between CpG treated B6 and SjS^S^ samples, reinforcing the PCA data (Fig. 1B). Similarly, the heat map indicates a divergent response to ACs between B6 and SjS^S^ BMDM samples, where more genes are upregulated in the SjS^S^ samples. This observation was confirmed in volcano plots depicting changes in gene expression control and AC treated conditions for B6 and SjS^S^ BMDMs. SjS^S^ BMDMs presented a stronger transcriptional response to ACs with 726 genes upregulated and 381 genes downregulated, compared to B6 BMDMs, which demonstrated 140 genes upregulated and 80 downregulated (Fig. 1C,D). In summary, these findings indicate that SjS^S^ BMDMs exhibit a transcriptional response to ACs that is distinct from that of B6 BMDMs and is differentiated by elevated gene expression.

### Inflammatory signaling pathways are upregulated in SjS^S^ BMDM following AC treatment

**Figure 2:**
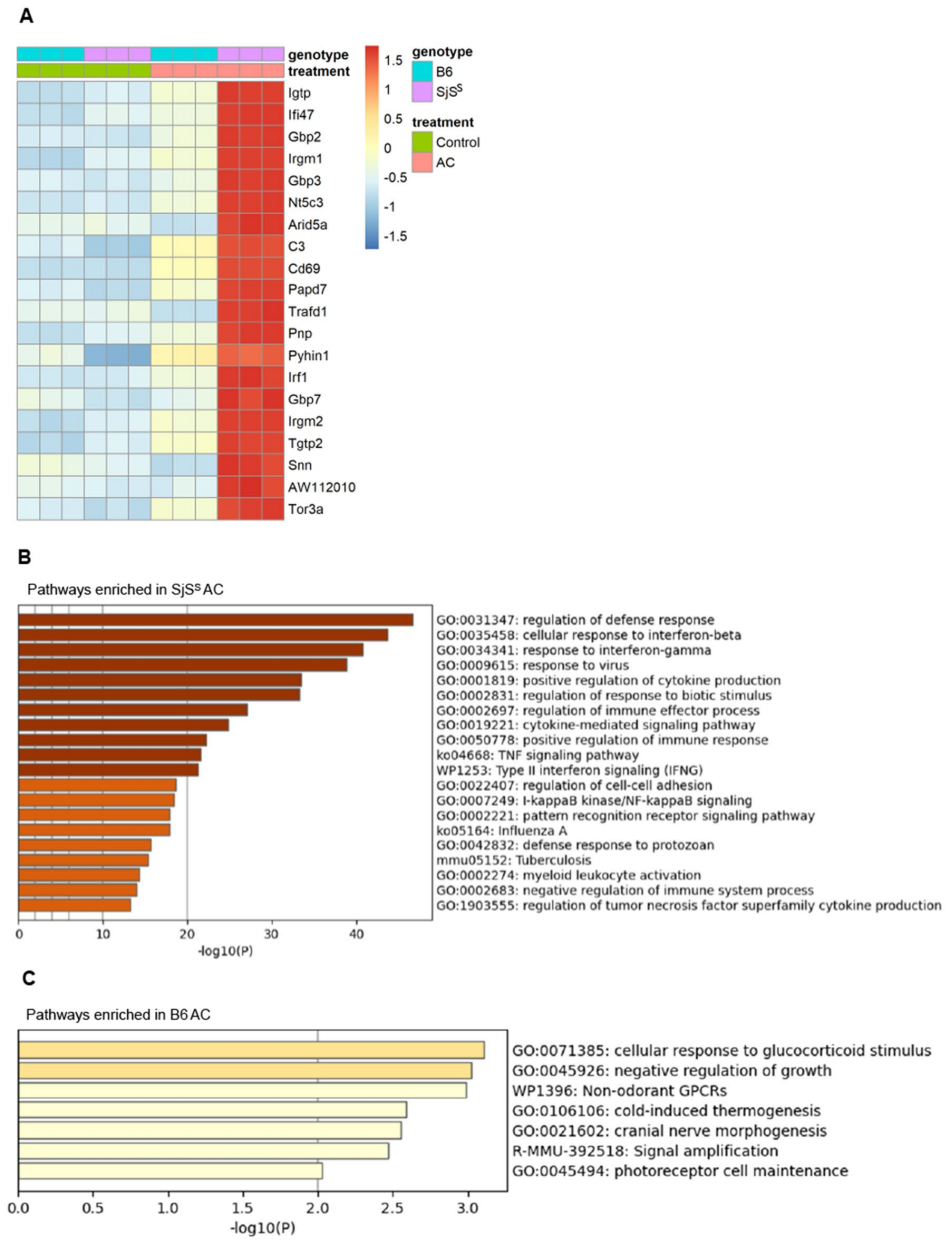
AC treated SjS^S^ BMDMs display an inflammatory transcriptional response. (**A**) Heat map depicting top 20 most highly upregulated differentially expressed genes in the AC SjS^S^ treatment condition with comparison to expression in other conditions (n = 3 mice per condition). (**B,C**) Enriched ontology clusters identified through Metascape revealed significant changes in gene pathways between SjS^S^ (**B**) and B6 (**C**) BMDMs after AC treatment.

Following the observation of a different response to ACs in B6 and SjS^S^ BMDMs, it was imperative to define the specific differences between the responses of these strains. As a starting point, the top 20 most highly upregulated genes were identified in the AC treated SjS^S^ population and included on a heat map against AC treated B6 BMDMs and control B6 and SjS^S^ samples (Fig. 2A). The 20 most upregulated genes in SjS^S^ BMDMs were primarily associated with inflammation (Fig. 2A). The most highly upregulated genes in AC treated SjS^S^ BMDMs primarily included genes related to IFN signaling (*Igtp*, *Ifi47*, *Gpb2*, *Gpb3*, *Irf1*, *Tgtp2*). However, genes related to macrophage activation (*Igrm1*, *Cd69*), cytokine and complement production (*Arid5a*, and *C3*), nucleic acid breakdown (*Pnp*), as well as regulators of the innate immune response (*Trafd1*) were also among the most highly upregulated genes by AC in SjS^S^ BMDMs. Importantly, none of these genes were upregulated in the AC-treated B6 BMDMs (Fig. 2A).

The observation of highly upregulated IFN signaling pathway genes in SjS^S^ BMDMs with AC treatment led us to perform Metascape pathway analysis on AC treated B6 and SjS^S^ BMDMs (Fig. 2B,C). In AC treated SjS^S^ BMDMs, pathways involving host defense were upregulated, involving pathways for tuberculosis, viral, and influenza A. IFNβ, IFNγ, and TNF pathways were all elevated, indicating overactivated inflammatory cytokine signaling. Pattern recognition receptor (PRR) pathway signaling, the NF-κB pathway, and myeloid cell activation pathways were all increased as well, suggesting that the inflammatory response to ACs mentioned above may be driven by enhanced SjS^S^ macrophage activation in response to ACs (Fig. 2B). Specific genes upregulated in the pathways for IFNβ response, IFNγ response, positive regulation of cytokine production, and cytokine mediated signaling pathway are collected in Supplementary Table 1. Supplementary Table 2 includes upregulated genes within the positive regulation of immune response, TNF signaling pathway, pattern recognition receptor signaling, and myeloid leukocyte activation pathways. As expected, B6 BMDMs stimulated with ACs did not exhibit the aberrant inflammatory response observed in SjS^S^ BMDMs, with no elevation in inflammatory pathways being detected (Fig. 2C). The stark difference in response to ACs between these two strains demanded further investigation, as efferocytosis is generally considered an immunologically silent process devoid of inflammatory signaling.

### SjS^S^ BMDM produce inflammatory cytokines following AC stimulation

**Figure 3:**
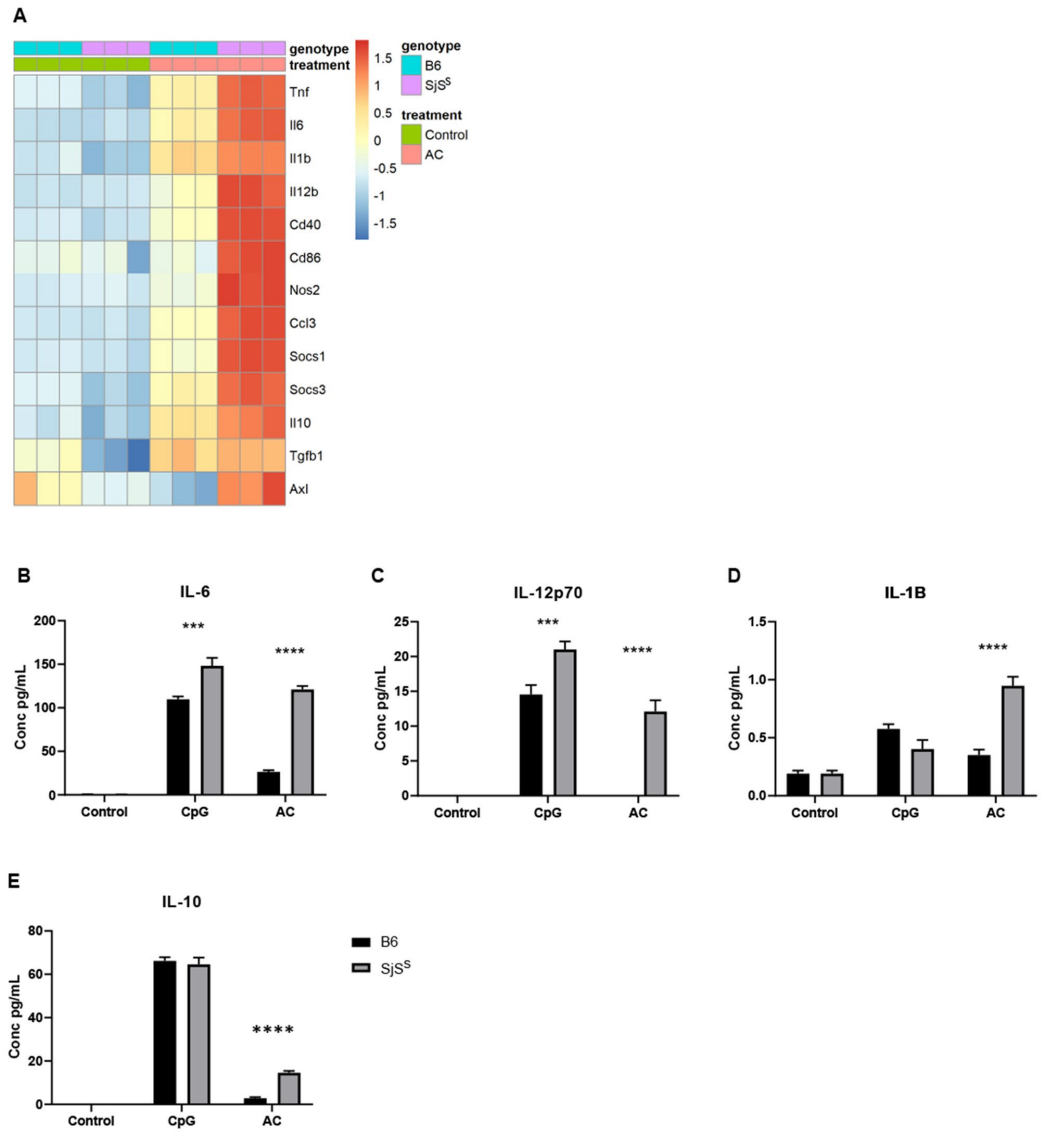
SjS^S^ BMDMs produce inflammatory cytokines in response to apoptotic cells. (**A**) Heatmap depicting RNAseq analysis results for differentially expressed selected inflammatory genes in B6 and SjS^S^ strains treated with Control, CpG, and AC conditions (n = 3 mice). (**B,E**) B6 (n = 3 mice) and SjS^S^ (n = 3 mice) BMDMs were treated with CpG 1 µM or AC for 24 h, at which point media was collected for Bio-plex cytokine analysis. The statistical significance was calculated by two-tailed unpaired *t*-tests where error bars indicate SEM ***p < 0.001, and ****p < 0.0001.

Considering that the top 20 most upregulated genes in AC treated SjS^S^ BMDMs included multiple genes related to IFN signaling and macrophage activation, and pathway analysis revealed the enrichment of numerous inflammatory pathways, RNA-Seq samples from this condition were further analyzed for expression levels of 12 specific inflammatory genes (Fig. 3A). AC treated SjS^S^ BMDMs were found to exhibit increased expression levels of acute-phase cytokines (*Il1b, Il6, Tnf), Il12b,* chemokines (*Ccl3*), co-stimulatory molecules (*Cd40, Cd86*), and markers of macrophage inflammatory state (*Nos2*). Of these genes, only *Il1b* showed a modest increase in AC-treated B6 BMDMs (Fig. 3A). The increase in chemokine and co-stimulatory molecule gene expression was especially interesting, as this represents a means of aberrant macrophage activity influencing the adaptive lymphocyte response. Lymphocytes are the primary components of the exocrine infiltrates in SjS, and this data indicates that atypical macrophage response could be a driver in downstream lymphocyte activation. Intriguingly, several anti-inflammatory genes were also upregulated by ACs in SjS^S^ BMDMs. *Socs1*, *Socs3*, *Il10*, *Axl*, and a moderate level of *Tgfb* were induced with ACs in SjS^S^ BMDMs while *Il10* and *Tgfb* were increased slightly in B6 BMDMs (Fig. 3A).

Inflammatory cytokine expression of AC treated SjS^S^ BMDMs was confirmed by a separate experiment measuring cytokine levels in the supernatant by Bio-Plex assay. In agreement with the RNA-Seq data, IL-6, IL-12p70, IL-1β were all found to be increased in AC treated SjS^S^ BMDM supernatant in comparison to AC treated B6 (Fig. 3B–D). Also, in accord with the RNA-Seq data, IL-10 was increased in the supernatant of AC treated SjS^S^ BMDMs over B6 BMDMs, possibly as a means to rein in the exaggerated inflammation through IL-6, IL-12p70, and IL-1β in this condition (Fig. 3E). Interestingly, increased IL-6 and IL-12p70 were detected in the supernatant of CpG treated SjS^S^ BMDMs over B6 BMDMs (Fig. 3B,C). This finding suggests that SjS^S^ BMDMs may have a stronger inflammatory response than B6 BMDMs following TLR engagement. However, according to the heat map and PCA plot generated above, the difference in response between B6 and SjS^S^ BMDMs to CpG appears to be less robust than the disparity in AC response by encompassing a smaller number of genes (Fig. 1A,B).

### Inflammatory response of SjS^S^ BMDMs to AC is abrogated by TLR inhibition

Next, it was imperative to recognize how ACs were driving the inflammatory response seen in SjS^S^ BMDMs. Interestingly, the Metascape pathway analysis had revealed that AC treatment activated PRR signaling pathways in SjS^S^ BMDMs (Fig. 2B). This observation was consistent with findings from previous studies indicating that, under conditions of impaired efferocytosis, self-antigens can leak out from apoptotic bodies and promote inflammatory signaling by activating TLR7 and TLR9^21^. Intriguingly, TLR7 was already a molecule of interest in SjS, as TLR7 is known to be upregulated in the submandibular gland of the SjS^S^ mouse before the onset of disease^22^. Additionally, TLR7 expression was reported to be increased in SjS patient peripheral blood mononuclear cells (PBMCs), and monocyte-derived dendritic cells from SjS patients exhibited elevated responsiveness to TLR7 activation^23,24^. Less is known about TLR9 in SjS, and it is unclear if it plays a protective or pathogenic function^25^. Therefore, the following experiment was conducted in order to address the role of TLR7 and TLR9 in facilitating the inflammatory response to ACs in SjS^S^ BMDMs.

Here B6 and SjS^S^ BMDMs were stimulated with TLR7 and TLR9 agonists (TLRa), ACs, or ACs with TLR inhibitors (TLRi) for TLR7 and TLR9. The objective was to determine if TLR inhibition could abrogate the SjS^S^ BMDM inflammatory response to ACs. TLR7 activation (TLR7a) was accomplished with 30ug/mL imiquimod, and TLR9a utilized 1 µM CpG. Both treatments proceeded for six hours. TLR7i incorporated 5 µM ODN 20958 and TLR9i involved 1 µM ODN 4084-F. TLRi treatments were initiated in BMDMs two hours before the addition of ACs and TLRi treatments were maintained during the two-hour incubation of ACs with BMDMs. Additionally, thymocyte apoptosis was accomplished by heat shock instead of dexamethasone treatment in order to establish an environment with more necrotic cell death and self-antigen leakage. RT qPCR was used to determine the expression of relevant inflammatory genes *Ifnb, Ifng, Il6, Il1b, Il12b, Il10, and Mer*.

**Figure 4:**
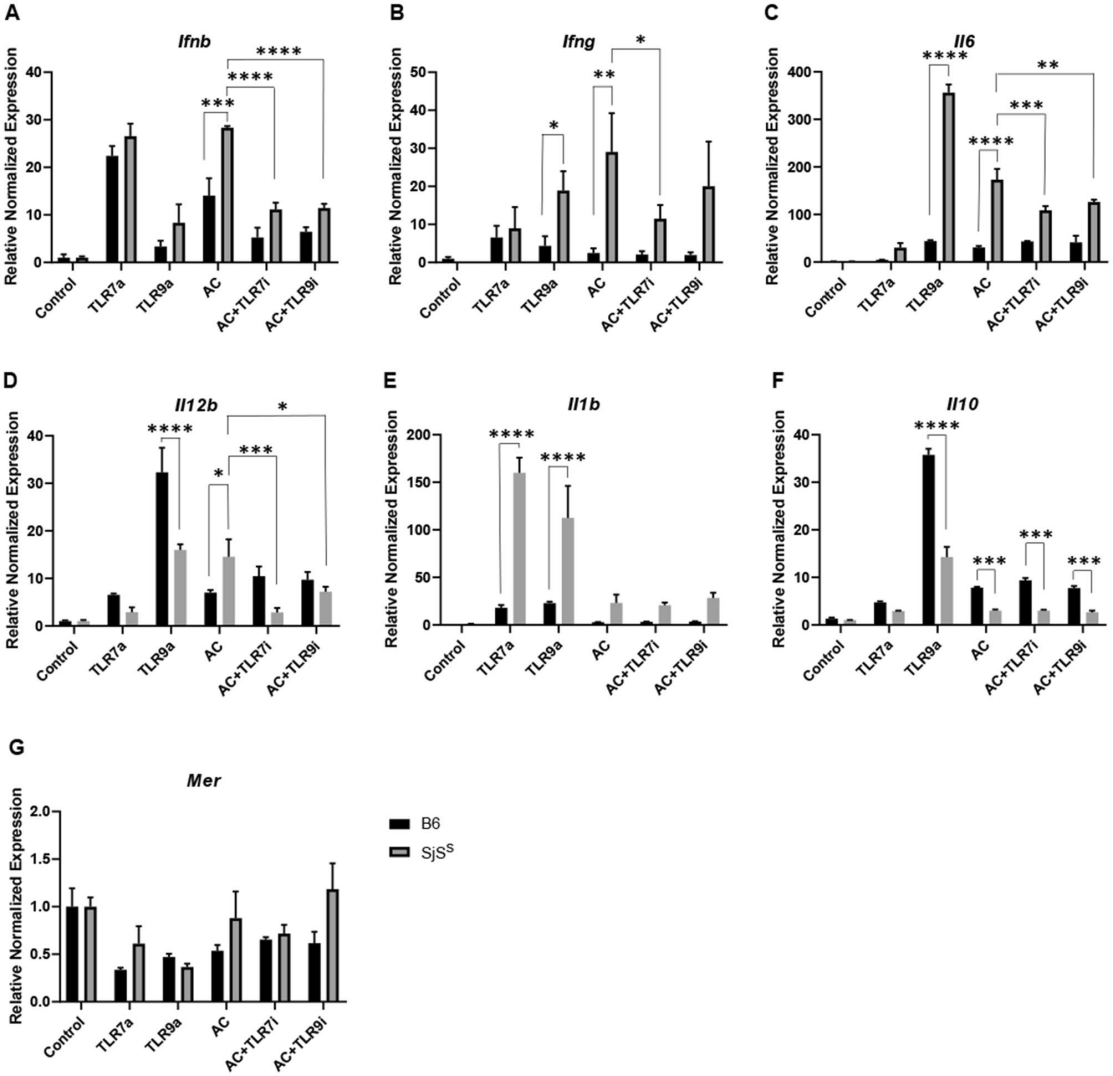
The inflammatory response of SjS^S^ BMDMs is mediated by TLR7 and TLR9. (**A,G**) BMDMs from B6 (n = 3 mice) and SjS^S^ (n = 3 mice) mice were treated with TLR7a (imiquimod) and TLR9a (CpG) to stimulate inflammation, AC, or AC plus TLR7i (ODN 20958), or AC plus TLR9i (ODN 4084-F) to inhibit TLR7 and 9 driven inflammation. All conditions were performed in triplicate. Upon conclusion of treatment, cells were washed, incubated for an additional three hours, and RNA was collected for RT qPCR. The statistical significance was calculated by one-way ANOVA where error bars indicate SEM *p < 0.05, **p < 0.01, ***p < 0.001, and ****p < 0.0001.

**Figure 5:**
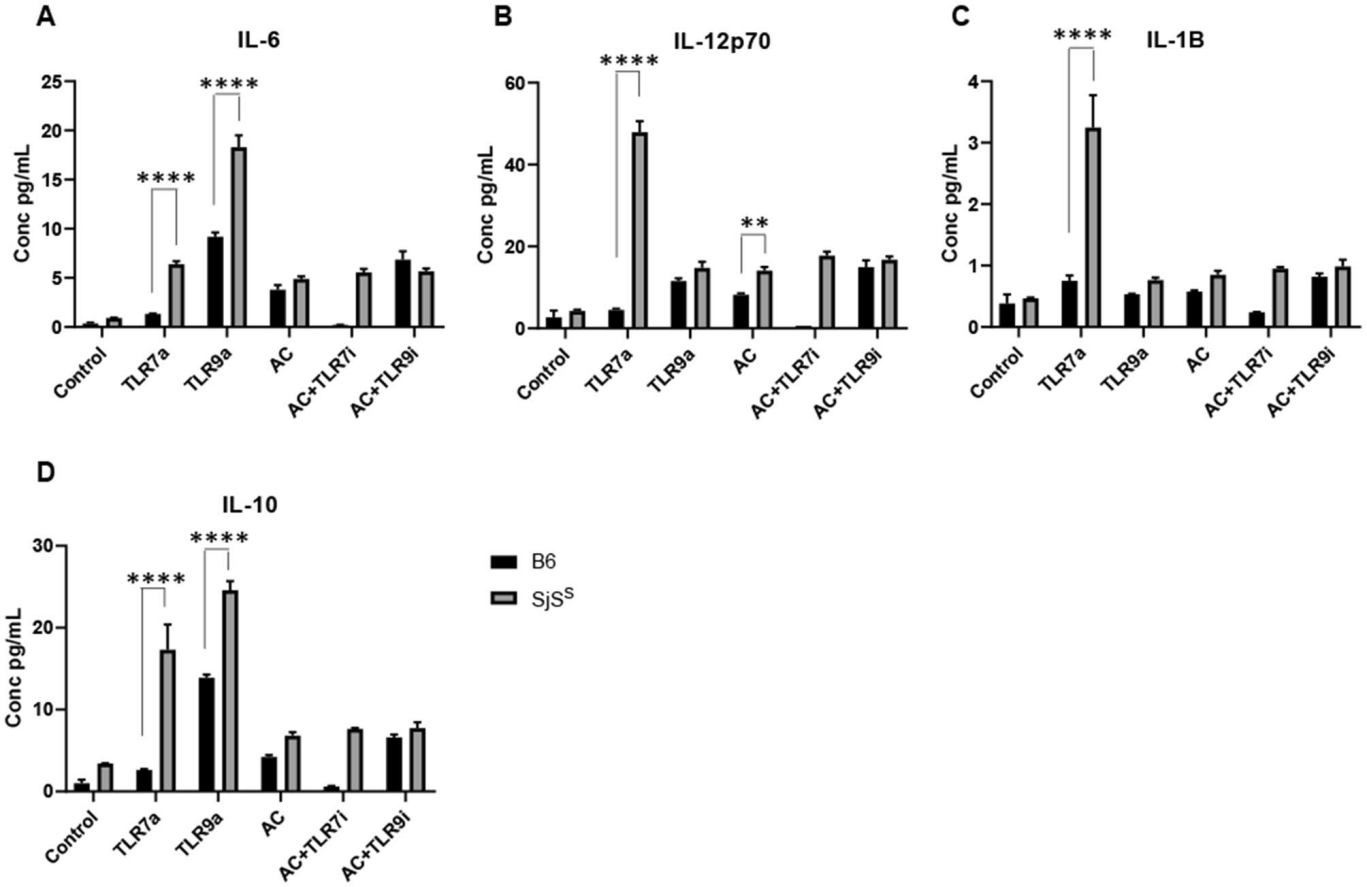
SjS^S^ BMDMs produce increased inflammatory cytokines in response to TLR stimuli. In Fig. 4 BMDMs from B6 (n = 3 mice) and SjS^S^ (n = 3 mice) mice were treated with TLR7a (imiquimod) and TLR9a (CpG) to stimulate inflammation, AC, or AC plus TLR7i (ODN 20958), or AC plus TLR9i (ODN 4084-F) to inhibit TLR7 and 9 driven inflammation. Cell culture supernatant was collected after a three hour incubation following conclusion of treatment and wash. (**A–D)** Cell culture supernatant was evaluated for cytokines with a Bio-Plex assay as performed in Fig. 3. The statistical significance was calculated by two-tailed unpaired *t*-tests where error bars indicate SEM **p < 0.01, and ****p < 0.0001.

*Ifnb* was induced by TLR7a at comparable levels in SjS^S^ and B6 BMDMs, whereas TLR9a activated only low levels of *Ifnb* expression (Fig. 4A). Consistent with the elevated IFN signaling pathways seen in the RNA-seq data, AC stimulation increased *Ifnb* expression in SjS^S^ BMDMs, and both TLR7i and TLR9i were capable of decreasing AC induced *Ifnb* expression (Fig. 4A). TLR9a induced *Ifng*, but only in SjS^S^ BMDMs (Fig. 4B). ACs did not increase *Ifng* in B6 BMDMs. This was in contrast to SjS^S^ BMDMs, where *Ifng* was highly elevated, though this increase was diminished by TLR7i (Fig. 4B). Both TLR9a and ACs increased *Il6* expression in SjS^S^ BMDMs, and both TLR7i and TLR9i reduced AC induced *Il6* expression in SjS^S^ BMDMs (Fig. 4C). Both *Il12b* and *Il1b* were more highly induced by AC treatment in SjS^S^ BMDMs than B6 BMDMs. However, this was only significant for *Il12b* (Fig. 4D,E). The modest induction of *Il1b* by ACs was not unexpected, as *Il1b* was only slightly upregulated in RNA-Seq and Bio-Plex data (Fig. 3A,D). Inhibition of both TLR7 and TLR9 was able to impair AC-induced *1l12b* expression in SjS^S^ BMDMs (Fig. 4D). Unexpectedly, *Il10* expression was higher in B6 BMDMs in all treatment conditions (Fig. 4F). Previously, *Il10* was slightly, but not significantly, elevated in B6 BMDMs over SjS^S^ BMDMs by CpG in our Bio-Plex data and more highly expressed in AC treated SjS^S^ BMDMs over B6 (Fig. 3A,E). This discrepancy in *Il10* induction between experiments is likely related to the heat-killed AC model used in this experiment. This finding suggests that SjS^S^ BMDMs upregulated *Il10* in response to apoptotic cells, but this effect is lost under conditions with more necrosis. No changes in expression of *Mer* were detected between strains regardless of treatment, consistent with our previous publication (Fig. 4G)^14^. Cell culture supernatant was collected from samples during the three-hour incubation following treatment and was evaluated for cytokine levels. While this short collection period was insufficient to fully capture the effects observable in the RT-qPCR data, the observation of enhanced sensitivity of SjS^S^ BMDMs to TLR7 and TLR9 ligands was reinforced by increased IL-6, IL-12p70, IL-1β levels (Fig. 5A–D). Taken together, these data reveal two separate and interrelated findings. Firstly, TLR engagement enhances the expression of multiple inflammatory cytokines (*Ifnb, Ifng, Il6, Il1b*) in SjS^S^ BMDMs over B6 BMDMs. Secondly, increased cytokine gene expression induced in SjS^S^ BMDMs by AC stimulation can be abrogated through inhibition of TLR7 or TLR9 signaling (*Ifnb, Ifng, Il6, Il12b*).

## Discussion

A recent study has described an increase in Mer inactivation contributing to impaired Mer signaling outcomes in SjS^S^ mice. The aim of this follow-up study was to determine if diminished Mer signaling altered macrophage response to apoptotic cells. Efferocytosis is considered to be an anti-inflammatory process. This is partially due to anti-inflammatory signaling mediated by Mer and partly due to the removal of dead cells, which can act as a source of inflammatory self-antigens. Consequently, we expected that apoptotic cells might fail to elicit anti-inflammatory signaling within the phenotype of increased Mer inactivation exhibited by SjS^S^ mice. Additionally, delayed disposal of dead cells was expected to permit the transition from apoptosis to secondary necrosis, allowing for TLR stimulation by the DAMPs, thus driving an inflammatory response.

To test this hypothesis, B6 and SjS^S^ BMDMs were incubated with ACs and ensuing transcriptional changes were evaluated via RNA-seq. SjS^S^ and B6 BMDMs exhibited a vastly divergent transcriptional response to ACs, with SjS^S^ BMDMs demonstrating more gene activation in general than B6 BMDMs and clustering separately by PCA analysis. Additionally, gene expression induced by ACs in SjS^S^ BMDMs was characterized by inflammatory pathways, and the majority of the top 20 most highly upregulated genes in this condition were related to inflammatory signaling, with an overrepresentation of genes related to IFN signaling. Acute phase cytokines, co-stimulatory molecules, and myeloid activation genes were all detected among the specific genes upregulated by AC treatment in SjS^S^ BMDMs.

The protein production of inflammatory cytokines was confirmed by measurement of IL-6, IL-12b, IL-1β, and IL-10 secreted into SjS^S^ BMDM supernatant at elevated levels following AC stimulation. Lastly, the effect of TLR inhibition on AC-induced inflammatory signaling was assessed in SjS^S^ BMDMs. Upon TLR stimulation, SjS^S^ BMDMs demonstrated increased expression of multiple inflammatory cytokines at levels in excess of what was observed in B6 BMDMs, suggesting an increased sensitivity to TLR stimulation according to Bio-Plex assay and RT qPCR data. Importantly, TLR7i and TLR9i were capable of diminishing AC-induced elevation in inflammatory genes in SjS^S^ BMDMs. This indicates that the inflammatory response to ACs in SjS^S^ BMDMs is mediated by the stimulation of TLR7 and TLR9 by AC-derived material.

Our findings are interesting in the context of recent studies describing increased TLR7 and TLR9 expression in primary SjS (pSjS) patient PBMCs and parotid glands and increased detection of IL-1β and TNF secreting cells in the pSjS peripheral blood; as our data indicate ACs can drive both TLR activation and cytokine expression in our SjS model^26,27^. A separate study from earlier this year also demonstrated that TLR7 expression is enhanced in SjS patient salivary gland tissue and was positively correlated with expression levels of *TNF*, *CXCL13*, *CXCR5* and *LT-α*^28^. Furthermore, this group indicated that TLR8KO mice develop SjS pathology that is driven by increased TLR7 signaling. TLR7/8KO abrogated this pathology which encompassed ectopic germinal centers, characteristic Ro and La autoantibodies, salivary gland infiltration, and inflammatory cytokine expression^28^. Interestingly, a 2018 study observed increased TLR7 expression on ductal cells and pDCs from labial salivary glands of SjS patients and speculated on the role of endogenous ssRNA as a driver of TLR7 activation and type I IFN signaling^29^. Monocytes from IFN positive SjS patients were found to have higher expression of TLR7, downstream signaling molecules MyD88, RSAD2 and IRF7, and cytoplasmic RNA sensing molecules RIG-I and MDA5 compared to controls^30^.

Interestingly, these findings are consistent with a previous study that assessed circulating plasmacytoid dendritic cells (pDCs) from SjS patients and non-SjS controls. The study observed that not only do pDCs from SjS patients exhibit a transcriptional signature indicative of endosomal TLR engagement, SjS pDCs produced more type IFNα and IFNβ when stimulated with TLR7 ligand loxoribine than pDCs from non-SjS controls^31^. Taken together, these results suggest that this phenotype of excessive inflammatory signaling among myeloid cells may be more pronounced in SjS than previously appreciated and appears to be shared among both macrophages and pDCs. It is also interesting to consider how the results of Kiripolsky et al. regarding the lack of SjS disease induction in NOD.B10^Myd88−/−^ mice fit into our model^32^. We found that TLR7 and 9 drove the inflammatory response of SjS^S^ BMDMs to ACs, indicating a specific role for MyD88 signaling downstream of AC recognition. Previous analysis of the NOD/Ltj mouse reported that PBMC TLR9 expression and p38 MAPK activation was increased from five to ten weeks old compared to controls, implying that TLR9 activation is an early event in SjS pathogenesis^33^. While our own results here cannot definitively establish temporality, we observed that AC-derived material stimulated TLR9 and upregulated inflammatory cytokines and genes for co-stimulatory molecules in SjS^S^ BMDMs, potentially initiating an environment supportive of lymphocyte activation and autoimmunity.

Increased detection of apoptotic cells and enhanced expression of pro-apoptotic genes have been reported in the salivary glands of both human patients and preclinical models^34–37^. While it has been posited that apoptotic cell material may be a driver of inflammation similar to SLE, the topic has been rarely studied in SjS^38^. An interesting 2017 study evaluated the response of the human pDC line GEN2.2 to apoptotic particles released from human salivary gland (HSG) cells. These particles were determined to contain SjS auto-antigens Ro, La, and α-fodrin, as well as single-stranded RNA. This group found that apoptotic particles from HSG cells activated TLR7 and TLR9, resulting in inflammatory cytokine release by pDCs^39^. A study by Wu et al. found that autoantigens including, topoisomerase I, poly (ADP-ribose) polymerase, U1-70 kd, and SSB/La were cleaved into fragments during apoptosis. Wu et al. observed that in addition to the leakage of intracellular antigens that occurs during secondary necrosis, autoantigens undergo additional post-translational modification during secondary necrosis, potentially enhancing their immunogenicity^40^.

Our study did not seek to identify the specific ligands released by the uncleared apoptotic cells that stimulated inflammatory signaling. Many necrosis related DAMPs have already been reported including “find me signals”, such as ATP, “eat me signals”, such as calcreticulin, and inflammatory molecules such as, heat shock proteins, high mobility box group protein 1 (HMGB1), uric acid, nucleic acids, ribonucleoproteins, and f-actin^41^. Many of these molecules have the capacity for stimulation of TLRs and other PRRs. Self-DNA and self-RNA are particularly interesting to consider, as they activate TLR9 and TLR7 respectively, and inhibition of these receptors was capable of abrogating apoptotic cell initiated inflammatory response. In a separate study, mice lacking expression of transcriptional regulator IκB-ζ experienced accelerated epithelial cell apoptosis and SjS symptoms, including serum antinuclear antibodies, lacrimal gland (LG) infiltration, and LG dysfunction^42^. This study by Okuma et al*.* implied that the phenotype of apoptotic cell accumulation in SjS could serve as a driver of autoimmunity and was not merely a consequence of the autoimmune attack. Our results further support this concept and provide a mechanism demonstrating how apoptotic cells are potent initiators of inflammatory signaling in SjS^S^ BMDMs.

Of the TAM receptors, Axl, but not Mer, or Tyro3 was found to be among the inflammation-induced genes in SjS^S^ BMDMs. This observation was not entirely surprising, as the role of Axl in moderating inflammation is well understood^43^. It is possible that anti-inflammatory signaling through Axl upregulated *Socs1, Socs3, Il10*, and to a lesser extent, *Tgfb* to dampen inflammation in AC treated SjS^S^ BMDMs. However, the data from RNA-Seq, Bio-Plex, and RT-qPCR indicating elevated expression of numerous inflammatory genes suggests that the induction of anti-inflammatory genes was inadequate to dampen the robust inflammatory response, at least by this time point. Curiously, enhanced SOCS3 expression has already been observed in SjS patient PBMCs and salivary gland tissue. The authors found that not only was SOCS3 expression enhanced, but it also failed to negatively regulate IL-6 induced pSTAT3, leading them to conclude that SOCS3 has reduced functioning in SjS^44^. Axl upregulation was not observed in B6 BMDMs, likely due to the absence of AC-induced inflammation in this condition. Our results also provide context for previous findings indicating that injection of a generalized inflammatory stimulus (Freund’s incomplete adjuvant) was sufficient to accelerate the onset of SjS symptoms in (NZB X NFZ)F1 mice^45^. We report that SjS^S^ BMDMs have an exaggerated inflammatory response coupled with insufficient negative regulation of inflammation, suggesting an increased susceptibility to inflammatory stimuli.

Taken together, these data demonstrate that SjS^S^ BMDMs exhibit an inflammatory response to ACs that is entirely different than what is seen in B6 BMDMs and is inconsistent with the anti-inflammatory nature of the efferocytic process. The presence of this phenotype in conjunction with the delayed efferocytosis already reported and the observation of the critical role of TLR engagement in this inflammatory response lends new support to the hypothesis that uncleared apoptotic cells maybe contributing to inflammation in SjS. Altogether, this data suggests that the delayed efferocytosis in AC treated SjS^S^ BMDMs resulted in the release of self-antigens, stimulation of TLRs, followed by upregulation of inflammatory genes.

## Supplementary Information

Supplementary Information.

## Data Availability

The datasets generated during the current study are available in the GEO repository, accession number GSE188734.
