# Supplementary material for: Bone marrow-derived macrophages from a murine model of Sjögren's syndrome demonstrate an aberrant, inflammatory response to apoptotic cells
Source: Sci Rep. 2022 May 21;12:8593. doi: 10.1038/s41598-022-12608-4 (PMC9124194; doi:10.1038/s41598-022-12608-4)
Supplement: Supplementary file 1 — Supplementary Information. [file 41598_2022_12608_MOESM1_ESM.pdf]

Supplementary Material

1 Supplementary Tables

Supplementary Table 1. Upregulated genes in AC treated SjS^{S} BMDMs within pathways identified in Figure 2B

Pathway	Input gene number/database gene numbers	Input genes
Cellular response to IFNβ	36/47	Gbp2b,Gbp2,Irgm1,Ifi203,Ifi204,Ifi47,Ifit1,Ifit3,Ifnb1,Igtp, Irf1,Acod1,Stat1,Tgtp1,Trex1,Irgm2,Gbp3,Iigp1,Pnpt1,Gbp6,Gm4841, Ifi207,Ifi205,Ifi209,Gm4951,F830016B08Rik,Ifi211,Aim2,Gm5431,Gm12185,Ifi213,9930111J21Rik1,Ifi208,Tgtp2,Mndal,Ifi206,Ifitm6
Response to IFNγ	52/137	Ciita,Socs1,Gbp2b,Gbp2,Gch1,H2-Aa,H2-Ab1,H2-Eb1,Irgm1,Cd74,Il12b,Il12rb1,Irf1,Acod1,Jak2,Gbp4,Mrc1,Nos2,Ccl12,Ccl2,Ccl22,Ccl3,Ccl4,Ccl5,Ccl7,Ccl8,Cx3cl1,Trim21,Stat1,Stxbp1,Tgtp1,Cd40,Zyx,Tlr2,Irgm2,Gbp3,Ccl24,Nmi,Kynu,Stx11,Gbp8,Parp9,Gbp6,Cdc42ep2,Il23r,Ifitm6, Gbp5,Gbp7,Gbp9,Nlrc5,Parp14,Gbp11
Positive regulation of cytokine production	78/452	C3,Casp4,Cd83,Camp,Cybb,F2r,F3,Flt4,Fzd5,Hpse,Ifi204,Cd74,Il10,Il12b,Il12rb1,Il15,Il18,Il1a,Il1b,Il6,Irf1,Jak2,Ltb,Mapkapk2,Mmp12,Myd88,Nr4a3,Eif2ak2,Ptgs2,Rasgrp1,Rel,Ccl2,Ccl3,Ccl4,Ccl5,Cx3cl1,Stat1,Tnf,Cd40,Tnfsf9,Tnfsf4, Clec5a,Tlr2,Klrk1,Icosl,Clec4e,Clec4n,Rsad2,Cd274,Rgcc,Peli1,Ifih1,Rnf135,Il33,Ulbp1,Zc3hav1,Dhx58,Tlr9,Nod1,Tlr3,Ripk2,Il23r,Il20rb,Cgas,Arid5a,Nlrp3,Ifi205,Gbp5,Ddx58,Malt1,Tarm1,Il27,Nod2,Tnfsf15,Scimp,Ifi211,Aim2,Mndal,Socs1,Dll1,Fcgr2b,Ifnb1,Acod1,Lag3,Gbp4,Nfkb1,Nos2,Pml,Srgn,Trim30a,Tnfaip3,Trex1,Axl,Macir,Nmi,Isg15,Cd86,Cfh,Ednrb,Gbp2,Gch1,Cxcl1,Cxcl10,Mrc1,Nfkbia,S100a9,Ccl12,Cxcl2,Slpi,Cmpk2,Irgm2,Cxcl11,Gbp6,Casp12,Fas,Gbp2b,Stat2,Tank,Gbp3,Nampt,Gbp7,Nox1,Daxx,Lpar1,Ldlr,Pim1,Dgat2,Insig1,Zfp366
Cytokine mediated signaling pathway	61/373	Casp4,Cd4,Socs1,Edn1,F3,Cxcl1,Irgm1,Cxcl10,Ifnb1,Cd74,Il12b,Il12rb1,Il13ra1,Il15,Il15ra,Il18,Il18rap,Il1a,Il1b,Il6,Irf1,Jak2,Lepr,Mmp12,Myd88,Nfkbia,Robo1,Ccl12,Ccl2,Ccl22,Ccl3,Ccl4,Ccl5,Ccl7,Ccl8,Cxcl2,Cx3cl1,Stat1,Stat2,Tnf, Traf1,Trex1,Ugcg,Klf6,Axl,Irgm2,Cxcl11,Ccl24,Zbp1,Iigp1,Nmi,Rps6ka5,Il33, Parp9,Ripk2,Il23r,Ifitm6,Il20rb,Aim2,Nlrc5,Parp14,Fas,Flt4,Il10,Il2ra,Kdr,Ltb, Kitl,Cd40,Tnfsf8,Tnfsf9,Tnfsf10,Tnfsf4,Xcr1,Ccrl2,Il21r,Tnfsf15,C3,Cflar,Epha4,F2r,Fcgr2b,Fgf1,Fpr2,Fzd5,Gcnt2,Lpar1,Htr2a,Icam1,Ighm,Mid1,Gadd45b,Eif2ak2,Rasgrp1,Trf,Gadd45g,Spry2,Tlr2,C1qtnf1,Cysltr2,Mlkl,Rapgef2,Tlr9,Nod1,Tlr3,Gpr55,Nox1,Tnfaip8l3,Nod2,Scimp,Glipr2,Cxadr,S100a9,Sell,Cd177,Rhoh,Slamf8,Jaml,Acod1,Nfkb1,Saa3,Tank,Dusp2,Dusp16,Wnk2,Aida,Spred1,Mapkapk2,Nrg1,Ch25h,Ednrb,Itga4,Mmp14,Ptpro,Vcam1,2610528A11Rik,P2ry12,Lrch1,Gch1,Dpysl2,Efna2,Fpr1,Kif5c,Lhx2,Nr4a3,B3gnt2,Flrt2,Gnb4,Adcy4, Alox12,Col18a1,Foxf1,Itgax,Ptgs2,Sod2,Tnfaip6,Cd274,Rin2,Rgs16,Asap3,Cdkl5

Supplementary Table 2. Continuation of upregulated genes in AC treated SjS^{S} BMDMs within pathways identified in Figure 2B

Pathway	Input gene number/database gene numbers	Input genes
Positive regulation of immune response	78/662	Bcl2a1d,C3,C4b,Cacnb3,Cd247,Cd4,Cfh,Fcgr1,Fcgr2b,Fpr2,Fpr1,Fzd5,H2-Ab1,Cfb,H2-M2,H2-T10,H2-T22,Irgm1,Ifi203,Ifi204,Ighm,Cd74,Il10,Il12b,Il12rb1,Il15,Il18,Il18rap,Il1b,Il6,Irf1,Acod1,Lag3,Lcp2,Mmp12,Nr4a3,Pnp,Rasgrp1,Stxbp1,Tnf,Cd40,Trex1,Tnfsf4,Usp12,Tlr2,Klrk1,Icosl,Irgm2,Rsad2,Zbp1,Cd274,Nmi,Rgcc,Cd177,Ifi35,Tasl,Il33,Ulbp1,Parp9,Nfkbiz,Tlr9,Ripk2,Il23r,Cgas,Arid5a,Nlrp3,Ifi205,Gbp5,Themis2,Ifi209,Malt1,Fyb2,Nod2,Ifi211,Aim2,Nlrc5,Ifi208,Mndal,Clec4e,Il20rb
TNF signaling pathway	32/107	Casp3,Casp7,Cflar,Socs3,Edn1,Fas,Cxcl1,Icam1,Cxcl10,Ifi47,Il15,Il1b,Il6,Mmp14,Nfkb1,Nfkbia,Ptgs2,Ccl12,Ccl2,Ccl5,Cxcl2,Cx3cl1,Tnf,Tnfaip3,Traf1,Vcam1,Map3k8,Rps6ka5,Mlkl,Creb5,Nod2,Gm5431,Bcl2a1a,Bcl2a1d,Ltb,Gadd45b,Myd88,Ccl4,Cd40,Trim25,Ddx58,Malt1,Lcn2,Mmp13,S100a9,Ccl7
Pattern recognition receptor signaling	34/157	Cd86,Irf1,Acod1,Mapkapk2,Myd88,Nfkbia,Trim30a,Tnf,Tnfaip3,Cd40,Tlr2,Irgm2,Clec4e,Rsad2,Nmi,Peli1,Ifi35,Tasl,Ifih1,Rnf135,Zc3hav1,Dhx58,Tlr9,Smpdl3b,Nod1,Tlr3,Ripk2,Lacc1,Tifa,Ddx58,Ddx60,Tlr11,Nod2,Scimp
Myeloid leukocyte activation	37/241	Slc7a2,Cst7,Ctsc,Dnase1l3,Fcgr2b,Fpr2,Foxf1,Ifnb1,Il10,Il15,Il18,Il18rap,Jak2,Lcp2,Ldlr,Myd88,Nr4a3,Rasgrp1,Ccl5,Cx3cl1,Stxbp1,Tnf,Tnfsf9,Trex1,Tlr2,Klrk1,Nampt,Nmi,Cd177,Ifi35,Batf2,Stx11,Rhoh,Il33,Ulbp1,Tlr9,Tlr3,Ptgs2,Nupr1,Cd200r4

2 Supplementary Figures

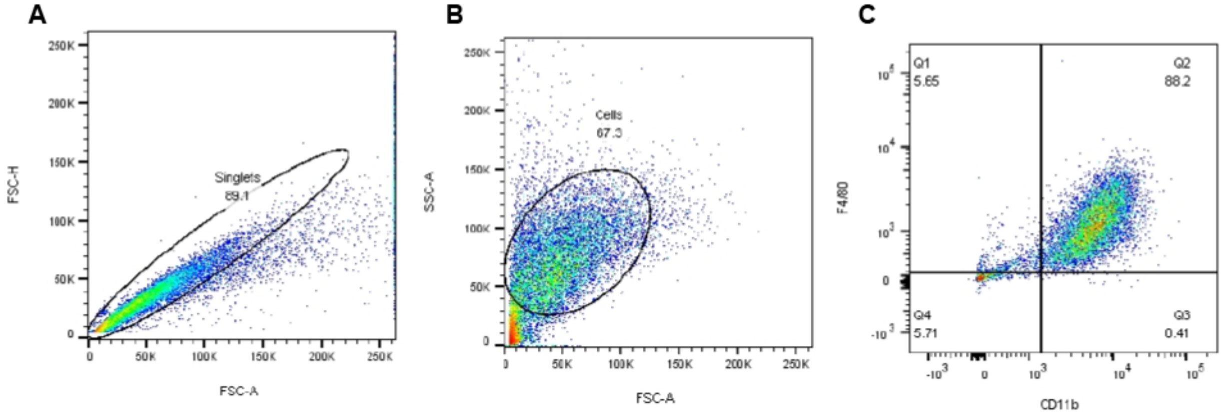

Supplementary Figure 1. Example of BMDM purity after seven days differentiation. Bone marrow cells were collected from femur and tibia from mice ranging from 7-12 weeks. Cells were differentiated into macrophages by seven days of incubation with media supplemented with 20% L929 supernatant. BMDM purity was assessed by flow cytometry at day 7. **A)** BMDMs were gated for singlets, **B)** FSC and SSC, **C)** approximately 90% of cells were double positive for macrophage markers CD11b and F4/80 by seven days.

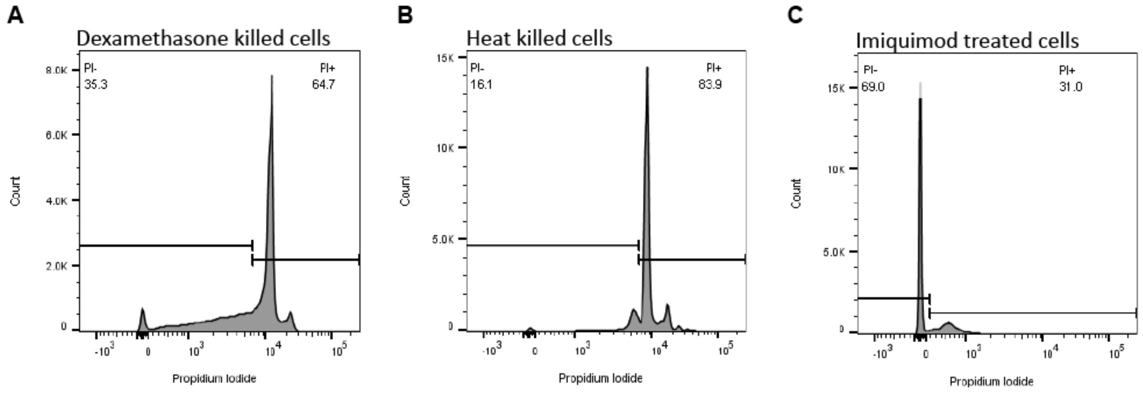

Supplementary Figure 2. Evaluation of cell death. A) Thymocytes were evaluated for propidium iodide positivity via flow cytometry following 6 hour treatment with 2μM dexamethasone. **B)** Similar analysis was performed on thymocytes killed by incubation for 20 minutes at 60°C. **C)** BMDMs were also evaluated for propidium iodide positivity following treatment with 30 μg/mL of imiquimod.
